# What Epigenetics Teaches Us About Neuron–Glioma Interactions

**DOI:** 10.1002/bies.70043

**Published:** 2025-07-20

**Authors:** Chaitali Chakraborty, Itzel Nissen, Silvia Remeseiro

**Affiliations:** ^1^ Department of Medical and Translational Biology Umeå University Umeå Sweden; ^2^ Wallenberg Centre for Molecular Medicine (WCMM) Umeå University Umeå Sweden

## Abstract

Neuron–glioma interactions are critical drivers of glioma progression, with neuronal activity promoting tumor growth and invasion through paracrine signaling and direct synaptic input. Beyond well‐established glutamatergic synapses, recent discoveries revealed that GABAergic interactions also contribute to glioma proliferation. Here, we focus on how glioma cells decode neuronal cues via epigenetic mechanisms, including enhancer reprogramming, chromatin remodeling, and rewiring of 3D genome organization, with transcriptions factors such as SMAD3 and PITX1 orchestrating transcriptional programs that sustain neuron‐to‐glioma communication. Additionally, recent integration of multi‐omics data highlights gene regulatory networks linked to GABAergic signaling as contributors to glioblastoma (GB) pathogenesis. We also underscore the distinct roles of GABAergic signaling across glioma subtypes, noting that, in GB, GABA‐related metabolic and paracrine mechanisms, rather than synaptic input, may drive tumor progression. Understanding how epigenetic reprogramming facilitates glioma integration into neural circuits opens new avenues to disrupt these malignant neuron–glioma interactions by targeting the epigenetic machinery.

## Graphical abstract

Neuron–glioma interactions through the epigenetic lens. Rewiring of epigenetic mechanisms, including alterations in the enhancer landscape and 3D chromatin organization, allows glioma cells to sense and respond to neural signals. Targeting the epigenetic machinery that facilitates this neuron–glioma crosstalk provides a strategy to disrupt the neural circuits sustaining glioma progression.

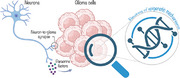

## 1 Introduction

Gliomas, particularly high‐grade gliomas such as glioblastoma (GB), exploit the neural environment to drive tumor progression. Far from being passive bystanders, neurons actively promote glioma growth, invasion, and therapy resistance through different mechanisms, including paracrine signaling and direct synaptic input [1, 2]. Neuronal factors like neuroligin‐3 (NLGN3) [3, 4, 5, 6], brain‐derived neurotrophic factor (BDNF) [3, 4], and insulin‐like growth factor‐1 (IGF‐1) [7] stimulate glioma proliferation. Additionally, functional glutamatergic neuron‐to‐glioma synapses further integrate tumor cells into active neural circuits, relaying electrical signals to glioma cells and thereby promoting tumor progression and invasion [5, 8, 9]. While glutamatergic neuron‐to‐glioma synapses have been described in GB and other high‐grade gliomas, only recent findings show that GABAergic synapses can also promote glioma proliferation in diffuse midline gliomas (DMG) [10].

A critical yet underexplored question is how glioma cells interpret and respond to these diverse neuronal signals at the genomic level. Epigenetic reprogramming emerged as a key mechanism in this decoding process [11, 12, 13]. GB cells exhibit extensive chromatin rewiring, reshaping the enhancer landscape and regulatory chromatin loops to sustain transcriptional programs that promote neuron–glioma interactions, with key transcription factors (TFs) like SMAD3 and PITX1 orchestrating these adaptations [11]. An epigenetically defined high‐neural signature, recently identified in GB, correlates with poor prognosis and enhanced neural connectivity [12]. Additionally, recent machine learning (ML)‐based integration of multi‐omics data uncovered regulatory networks linked to GABAergic pathways as relevant in GB pathogenesis, suggesting a broader range of neuron–glioma communication mechanisms in high‐grade gliomas [13].

Here, we focus on how neuron–glioma interactions begin to be decoded through the epigenetic lens, highlight the emerging insights into GABAergic signaling in high‐grade gliomas, and offer new perspectives for targeting the epigenetic machinery to disrupt the neuron–glioma interactions driving tumor progression.

## 2 Neuron–Glioma Interactions in Tumor Progression

**FIGURE 1:**
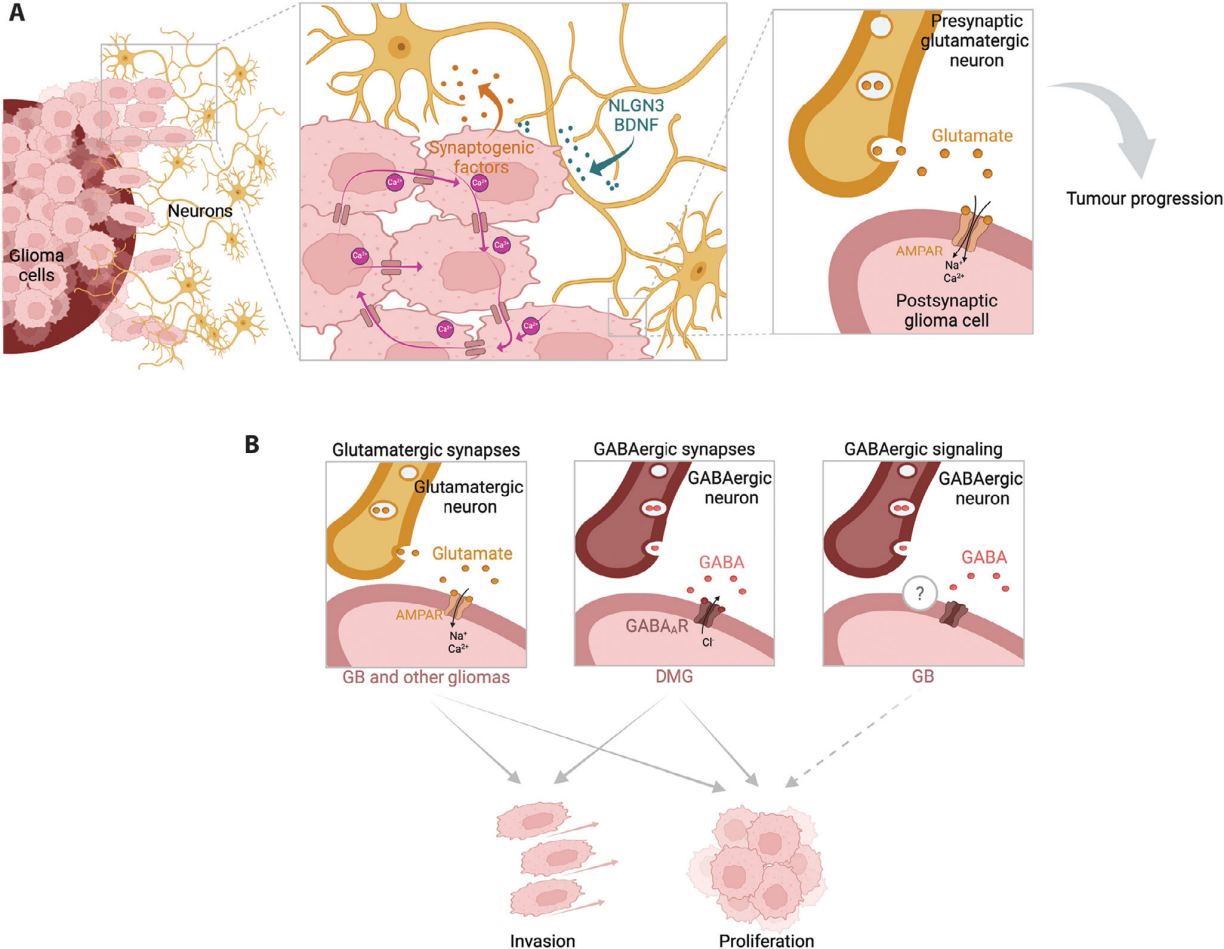
**Neuron–glioma interactions in the central nervous system drive tumor progression**. **(A)** Neuronal activity drives glioma growth through paracrine signals (e.g., NLGN3, BDNF), and by electrochemical communication mediated by glutamatergic neuron‐to‐glioma synapses involving AMPA receptors. Depolarizing currents propagate through gap junction‐coupled glioma networks, amplifying these currents within the network, and promoting glioma cell proliferation. Glioma cells also release glutamate and synaptogenic factors (e.g., glypican‐3, TSP‐1), enhancing neuronal excitability in the tumor microenvironment, which thereby further promote tumor progression and invasion. **(B)** Tumor‐promoting roles of glutamatergic and GABAergic signaling in gliomas: (Left) In glioblastoma (GB) and other high‐grade gliomas, glutamatergic neuron‐to‐glioma synapses using AMPA receptors deliver excitatory synaptic input, inducing membrane depolarization and promoting tumor progression and invasion. (Center) In diffuse midline gliomas (DMG), synaptic input from GABAergic interneurons via GABA_A_ receptors similarly drives membrane depolarization and tumor progression. (Right) In GB, co‐culture assays have shown that GABAergic signaling promotes GB cell proliferation through non‐synaptic mechanisms, likely involving metabolic or paracrine interactions. Created with BioRender.

The nervous system has emerged as a key regulator of cancer, including gliomas. One way in which neuronal activity can modulate glioma biology is through paracrine signaling mechanisms that release soluble growth‐promoting factors (Figure 1A). Among these, NLGN3 plays a pivotal role in high‐grade [3, 4, 5] and low‐grade [6] glioma proliferation. BDNF and glucose‐regulated protein 78 (GRP78) also contribute to this pro‐tumorigenic environment in gliomas [3, 4], while IGF‐1 drives the growth of olfactory bulb glioma [7]. Additionally, neuronal activity in brain regions contralateral to the primary tumor promotes glioma cell migration via semaphorin 4F (SEMA4F), demonstrating how remote neuronal cues can orchestrate tumor spread [14].

In a groundbreaking discovery, glioma cells were also shown to integrate into neural circuits by forming glutamatergic synapses with neurons, thereby contributing to tumor growth and invasion [5, 8, 9] (Figure 1A). Through these synapses, glioma cells receive excitatory synaptic input predominantly via calcium‐permeable AMPA receptors, inducing membrane depolarization, intracellular calcium influx, and activation of pathways that drive tumor progression and invasion [5, 8, 9]. Both pharmacological and genetic inhibition of AMPA receptors reduce glioma growth and improve survival in preclinical models [5, 8, 9]. Notably, clinical trials have been initiated to test the effect of perampanel, an antiepileptic AMPA receptor inhibitor, in patients with progressive GB [15].

Additionally, a notable link exists between paracrine and synaptic mechanisms. NLGN3 induces a synaptogenic gene expression profile in glioma cells, suggesting it may act as an upstream regulator of malignant synaptogenesis [4]. BDNF also promotes synaptic connectivity between neurons and glioma cells by increasing AMPA receptor trafficking, thereby influencing the strength of these malignant synapses [16].

Furthermore, a subset of glioma cells, interconnected through tumor microtubes (TMs) via gap junctions [17], form glioma‐to‐glioma networks and respond to neuronal activity with potassium‐evoked currents [5, 8]. The glioma cell networks also exhibit autonomous rhythmic activity, generated by pacemaker‐like tumor cells located at functional hubs within the network [18]. These rhythmic tumor cells influence the other cells in the network by generating intercellular calcium waves, which propagate throughout the network, driving brain tumor progression [18], and enhancing glioma resistance to chemotherapy and radiotherapy [19, 20].

Beyond glutamatergic signaling, recent studies point to particular neural interactions in specific glioma subtypes. For instance, some pediatric‐type high‐grade gliomas appear to rely more on GABAergic signaling. Diffuse hemispheric gliomas H3G34 mutant (DHG‐H3G34), which originate from interneuronal precursors, exhibit a GABAergic interneuronal‐lineage identity, and recapitulate the spatial and phenotypic features of early GABAergic interneuron development [21]. Functional GABAergic synapses have been identified in DMG, where GABAergic interneurons form functional synapses with glioma cells via GABA_A_ receptors [10] (Figure 1B). In this scenario, the GABAergic signaling has a depolarizing effect on glioma cells, which enhances tumor proliferation in DMG [10].

In addition to responding to neural cues, glioma cells actively remodel their microenvironment by secreting synaptogenic factors such as glypican‐3 [22, 23] and TSP‐1 [24], which promote neuronal hyperexcitability and functional reorganization of neural circuits (Figure 1A). Such increased neuronal activity in the tumor microenvironment, in turn, further drives glioma progression.

This integration of synaptic, paracrine and network‐based interactions between neurons and glioma cells highlights the intricate, complex and dynamic nature of the mechanisms driving brain tumor progression. Insights into neuron–glioma interactions are now advancing from discovery to clinical trials, marking a new phase in glioma therapy development.

## 3 From Circuitry to Chromatin: Unraveling Epigenetic Control of Neuron–Tumor Interactions in Glioblastoma

Neuronal activity drives targeted epigenetic remodeling that facilitates synapse formation and neural circuit development in the healthy brain. HDAC2 has been identified as a negative regulator of synaptic plasticity and memory establishment [25]. Its inhibition promotes dendritic spine density, synapse number, memory formation, and synaptic plasticity, highlighting the role of histone acetylation in activity‐dependent circuit remodeling [25]. Moreover, neuronal activity is directly coupled to gene induction. Different patterns of neuronal stimulation trigger specific waves of activity‐regulated gene expression, revealing a finely tuned transcriptional program that links the duration of neuronal activity to defined synaptic outcomes [26]. The development of dendritic complexity also depends on neuron‐specific chromatin remodeling complexes (nBAF), which interact with activity‐responsive regulators such as CREST to control gene expression required for dendritic growth [27]. At the genomic level, activity‐dependent enhancers (enriched for H3K4me1, bound by CBP, and showing increased H3K27ac following membrane depolarization) regulate gene expression programs critical for neural development and function [28]. These tightly regulated epigenetic mechanisms that govern neural circuit formation in the healthy brain appear to be hijacked in gliomas, which exhibit widespread epigenetic alterations and exploit neuron–glioma interactions to promote tumor progression.

### 3.1 Epigenetic Features of Gliomas

Epigenetic dysregulation in gliomas encompasses a wide range of processes including DNA methylation, histone modifications, chromatin accessibility, and 3D genome architecture. Changes in the epigenetic landscape play a central role in the aetiology of gliomas and are used for molecular classification [29]. The CpG island methylator (G‐CIMP) phenotype, often linked to *IDH1* mutations, is defined by extensive DNA hypermethylation and correlates with improved glioma prognosis [30]. A paramount example of an epigenetic marker used clinically for diagnosis is the *MGMT* promoter hypermethylation, which predicts response of GB tumors to the alkylating agent temozolomide [31, 32]. In GB, initial studies identified four subtypes – classical, mesenchymal, neural, and proneural – based on distinct aberrations and gene expression patterns [33]. More recently, single‐cell analyses have revealed that GB cells exhibit a high degree of plasticity, transitioning between multiple cellular states shaped by the tumor microenvironment [34]. Additionally, glioblastoma stem cells (GSCs) display a continuum of developmental and injury‐response transcriptional programs [35].

Over the past years, efforts to integrate transcriptomic data with chromatin accessibility, DNA methylation, and 3D genome architecture have further highlighted the molecular complexity and distinctiveness of GB compared to low‐grade gliomas. In high‐grade gliomas, mutations in chromatin remodelers and epigenetic‐related enzymes lead to widespread changes in gene expression [30, 36, 37]. Histone modifications are frequently altered, affecting the expression of genes relevant for gliomagenesis. Mutations in *H3F3A* and *HIST1H3B* – encoding histones H3.3 and H3.1, respectively – are found in gliomas, particularly in pediatric high‐grade gliomas and diffuse intrinsic pontine gliomas (DIPGs) [38, 39]. These histone mutations disrupt normal methylation patterns, especially at H3K27me3 and H3K36me3, contributing to gliomagenesis [36]. In particular, the H3K27M mutation reduces global H3K27me3, promoting oncogenic gene expression and defining a highly aggressive glioma subtype [40, 41]. Moreover, overexpression of the chromatin remodeler LSH promotes GB progression [42], while various non‐coding RNAs influence gene networks involved in metabolism and tumor growth [43, 44]. Chromatin structural variation adds another layer of complexity. Studies using chromosome conformation capture techniques such as Hi‐C have revealed substantial alterations in genome organization in GB, with translocations, inversions, deletions, and duplications, alongside the formation of neo‐TADs (Topologically Associating Domains) and neo‐loops leading to ectopic enhancer‐promoter contacts [45, 46]. These findings highlight the extensive heterogeneity of 3D genome organization across GB patients. Additionally, mapping active enhancers and promoters in benign and malignant gliomas revealed the relevance of the FOXM1‐ANXA2R regulatory axis in gliomagenesis [47]. Developmentally encoded transcriptional states in GB are linked to two main super‐enhancer profiles, and *SOX2* activation, supported by promoter hypomethylation and H3K27ac enrichment, helps sustain the GSC state [48]. Furthermore, ChRO‐seq (Chromatin Run‐On and Sequencing) revealed how TFs drive coordinated gene expression, contributing to epigenetic heterogeneity in GB tumors [49]. Altogether, this highlights the central role of epigenetic regulation in shaping glioma heterogeneity and malignant behavior.

### 3.2 Epigenetic Regulation Underlying Neuron–Glioma Communication

Although it has been known for some years that glioma cells respond to paracrine signals and form glutamatergic synapses with neurons to drive proliferation and invasion [3, 5, 8, 9], the question of how these external neural signals are decoded at the genomic level by glioma cells has only recently begun to be addressed.

**FIGURE 2:**
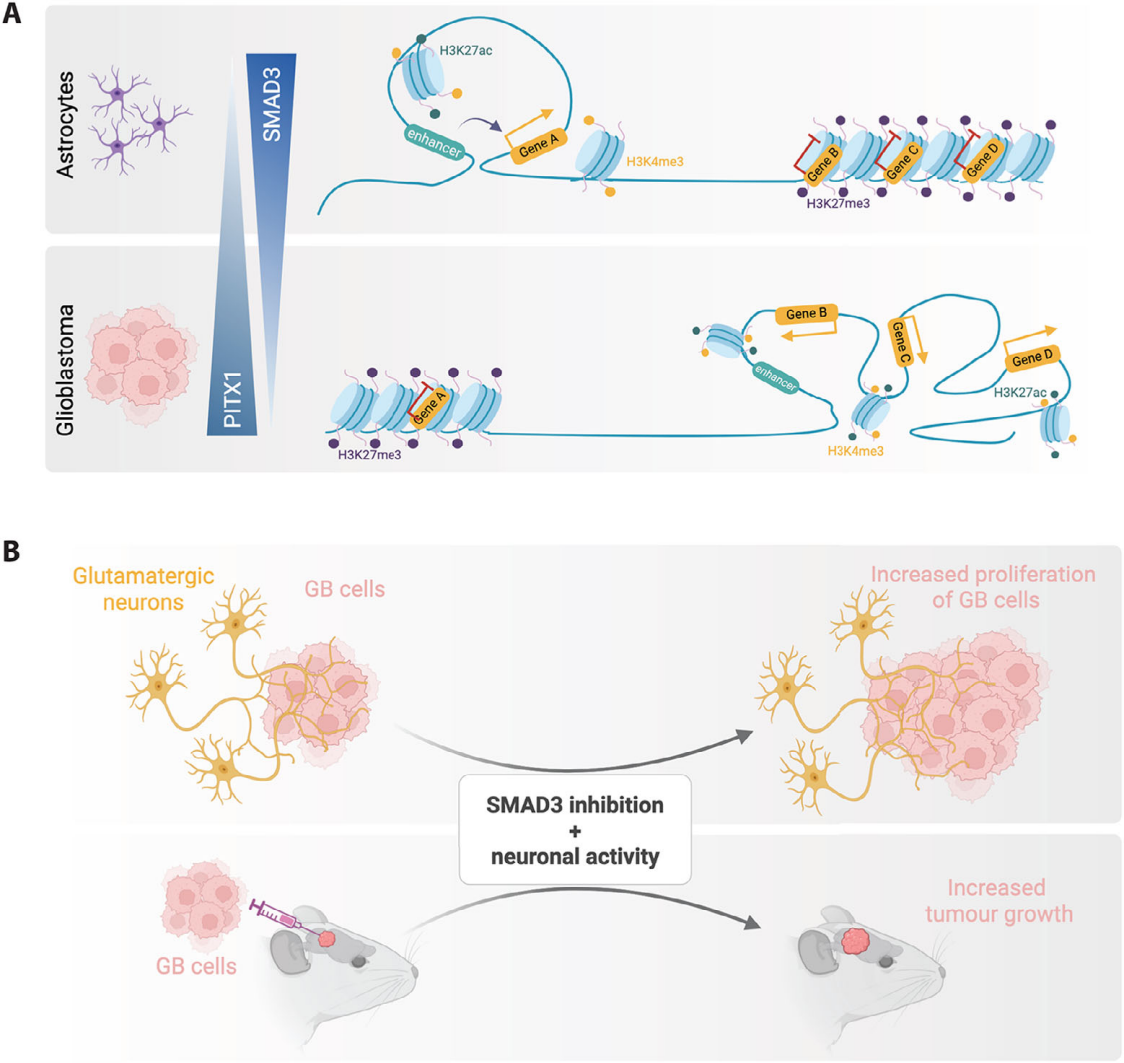
**Chromatin rewiring sustains transcriptional programs enabling neuron‐to‐glioma synaptic communication in glioblastoma (GB). (A)** In GB, the enhancer landscape and 3D genome architecture are rewired, with loss of long‐range loops and gain of shorter promoter‐promoter interactions. These changes are accompanied by altered patterns of histone modifications and chromatin accessibility, reshaping gene expression programs linked to glutamatergic synapses, axon guidance, and axonogenesis. Transcription factors SMAD3 and PITX1 are key direct regulators of a subset of these genes. **(B)** SMAD3 inhibition and neuronal activity cooperate to promote the proliferation of GB cells both in vitro in co‐culture assays, and in vivo in patient‐derived orthotopic models. Created with BioRender.

Our group recently mapped a rewiring in the epigenetic and 3D regulatory landscape that sustains neuron‐to‐glioma synaptic communication in GB [11]. Using an integrative multi‐omics approach (which included RNA‐seq, ATAC‐seq, ChIP‐seq for H3K27ac, H3K4me3 and H3K27me3, as well as H3K4me3 HiChIP), we characterized profound alterations in the enhancer landscape and promoter‐enhancer interactome in 15 patient‐derived GB lines. The rewiring observed was featured by the loss of long‐range regulatory interactions and the gain of shorter‐range promoter‐promoter loops, indicating a topological reorganization of the genome that favors tightly clustered promoter hubs [11]. These structural changes were accompanied by altered patterns of histone modifications and chromatin accessibility, with loss of active enhancer marks at particular chromatin states and activation of gene promoters. Altogether, these alterations orchestrate changes in gene expression that support neuron–glioma communication, particularly in genes involved in glutamatergic synapses, axon guidance, axonogenesis, and chromatin remodeling [11] (Figure 2A).

Furthermore, our study uncovered the TFs SMAD3 and PITX1 as major direct regulators of a set of target genes related to synapse organization, glutamatergic synapses, and axon guidance in GB [11]. Their binding motifs were enriched in active chromatin regions gained in GB and at the anchors of differential chromatin loops, and genome‐wide binding analysis confirmed that SMAD3 and PITX1 occupy the promoters of certain genes involved in synaptic communication, whose expression changes in response to modulated levels of these TFs [11]. Importantly, SMAD3 inhibition and neuronal activity stimulation cooperate to promote the proliferation of GB cells both in vitro, in co‐culture with human glutamatergic neurons, and also drive tumor progression in vivo, in patient‐derived orthotopic xenograft models [11] (Figure 2B). These findings not only established a mechanistic link between neural input and tumor growth, but also demonstrated that epigenetic regulators of this neuron‐to‐glioma axis can serve as actionable targets.

Additional recent studies also highlight distinct epigenetic mechanisms that may facilitate neuron–glioma interactions. In *IDH‐*wildtype GBs carrying RTK (receptor tyrosine kinase) mutations, mTORC2 drives genome‐wide DNA hypomethylation by epigenetically silencing *DNMT3A* via EZH2 recruitment to its promoter [50]. This altered RTK‐mTORC2 signaling epigenetically remodels a glutamate metabolism network to support GB cell survival, and potentially affecting neuron–glioma interactions [50]. Widespread miRNA dysregulation in GB is characterized by the downregulation of miRNAs linked to tumor microenvironment and extracellular matrix remodeling, while upregulated miRNAs are strongly associated with neural functions, including genes involved in synaptic communication [51]. Moreover, an epigenetically defined high‐neural signature has been identified in GB, exhibiting hypomethylated CpG sites and increased expression of synaptic integration genes [12]. This signature predicts poor patient survival and is associated with enhanced neural lineage features, synaptic integration, and functional brain connectivity, underscoring its prognostic and therapeutic significance [12].

Together, these findings highlight that epigenetic rewiring enables GB cells to integrate and respond to neural cues, setting the stage for the discovery of additional gene regulatory networks (GRNs) – beyond the ones identified in Ref. [11] – that mediate and diversify neuron–glioma communication. Building on this, our most recent study, employing an ML‐based integration of multi‐omics data, identified additional GRNs linked to not only well‐established glutamatergic pathways, but more importantly, to GABAergic signaling, which had not been previously implicated in GB pathogenesis [13]. These networks capture genes encoding for GABA receptors, ion channels involved in neurotransmission, TFs involved in neural differentiation and regulation of GABAergic neurons, alongside other genes such as *GAD1*, encoding the enzyme catalyzing the synthesis of GABA [13]. TFs known for their role in GABAergic neuron differentiation – including ARX, GSX2, and DLX family members – were identified as key regulators in these GB‐relevant networks [13]. These TFs drive expression of genes related to GABA signaling and are themselves associated with distinct chromatin patterns, suggesting epigenetic control of GABAergic features in GB. Furthermore, co‐culture assays revealed that both glutamatergic and GABAergic neurons increased the proliferation of GB cells, pointing to a novel role for GABAergic signaling in GB progression [13] (Figure 1B).

Epigenetic and chromatin rewiring is therefore positioned as a key mediator of neuron–glioma crosstalk. Glioma cells adapt and respond to neuronal cues by reconfiguring their transcriptional and chromatin landscapes to promote integration into neural networks [11, 12, 13]. Notably, modulation of specific TFs involved in neuron‐to‐glioma synaptic communication has been shown to influence tumor growth [11]. Thus, targeting the epigenetic machinery that facilitates this neuron–glioma interaction offers a strategy to disrupt the neural circuitry sustaining GB progression.

## 4 New Perspectives on GABAergic Signaling in High‐Grade Gliomas

Neuron–glioma interactions are critical for glioma progression, with glutamatergic synaptic signaling having a well‐established role [5, 8]. By contrast, the contribution of GABAergic signaling has remained less clear until recently, when some studies have revealed novel roles for GABAergic input in gliomas. Notably, functional GABAergic synapses have been identified in DMG, a type of pediatric high‐grade glioma, where GABAergic interneurons form synaptic connections with glioma cells through GABA_A_ receptors [10]. Unlike their typical inhibitory role in the mature healthy brain, GABAergic input in DMG induces membrane depolarization due to elevated intracellular chloride levels, ultimately promoting tumor proliferation [10]. Moreover, increasing GABAergic signaling with benzodiazepines like lorazepam accelerates tumor growth, whereas GABA_A_ receptor antagonism reduces it. These findings reveal that, at least in this specific glioma subtype, GABAergic signaling can exert a depolarizing tumor‐promoting effect. However, the scenario appears different in hemispheric high‐grade gliomas such as *IDH‐*wildtype GB, which seem to exhibit only minimal depolarizing GABAergic currents [10]. Nevertheless, transcriptome profiling shows that diffuse hemispheric gliomas carrying H3G34 mutations (DHG‐H3G34), adopt GABAergic interneuron‐like identity [21]. This suggests that elements of GABAergic signaling may be retained or co‐opted at a molecular level, even when synaptic GABAergic currents are absent. Indeed, genes encoding GABA receptors are expressed in patient‐derived GB lines, even though to a lower level than AMPA receptor genes [11]. The observation that GB cells display transcriptional programs resembling GABAergic interneurons [21] is particularly intriguing in light of recent finding showing that a specific population of interneuron progenitors has been shown to give rise to brain tumors [52].

Applying the ML‐based MOBILE pipeline to multi‐omics datasets, we identified regulatory networks linked to GABA signaling as relevant for GB pathogenesis [13]. Our co‐culture assays showed that GB cell proliferation increases not only in the presence of glutamatergic neurons, but also when exposed to GABAergic interneurons [13]. While the proliferation‐promoting effect of glutamatergic neurons on GB cells aligns with previous findings [5, 8], our co‐culture experiments unexpectedly revealed that GABAergic neurons also enhance GB cell proliferation. Although the effect of glutamatergic neurons was stronger, suggesting a more prominent role for glutamatergic inputs, GABAergic interneurons also contributed significantly to increased GB cell proliferation. However, their modes of action and underlying mechanisms appear to differ. Blocking AMPA receptors with NBQX reduced the proliferation of GB cells in co‐culture with glutamatergic neurons [13], aligning with the tumor‐promoting role of glutamatergic synapses [5, 8]. However, pharmacological blockade of GABA_A_ receptors with picrotoxin had no effect on GB cell proliferation in the co‐culture with GABAergic interneurons [13]. This suggests that, in GB, GABAergic signaling operates primarily through non‐synaptic mechanisms, likely promoting GB proliferation through metabolic or paracrine interactions rather than via direct synaptic depolarization (Figure 1B). In fact, beyond synaptic communication in DMG, GABAergic signaling plays other roles in brain tumors. GABA serves as a nutrient in medulloblastoma, where it is metabolized to support tumor growth [53], and inhibition of GABA catabolism using vigabatrin reduces brain metastasis by limiting the availability of key metabolic intermediates to tumor cells [54]. These observations introduce the idea that GABA not only acts as a neurotransmitter but also as an important metabolic fuel in the tumor microenvironment. Therefore, in GB, GABAergic signaling may be exploited both to fuel metabolic process using glutamine through increased *GAD1* expression, and to promote oncogenic neuron–glioma interactions.

Altogether, these emerging insights suggest that GABAergic signaling in high‐grade gliomas is context‐dependent (Figure 1B). In certain subtypes, such as DMG, direct GABAergic synaptic communication has been shown to drive tumor progression [10]. By contrast, in GB, while the presence of GABAergic synapses might not be fully ruled out yet, they do not appear to play a major role. Instead, the effect of GABAergic signaling in GB proliferation may primarily operate through metabolic or paracrine mechanisms rather than direct synaptic transmission [13]. Understanding these subtype‐specific differences in GABAergic involvement could reveal new vulnerabilities and therapeutic opportunities.

## 5 Conclusions

Neuron–glioma interactions have emerged as critical regulators of glioma progression, changing our understanding of how tumors exploit their neural environment [1, 55]. Neuronal activity promotes glioma growth through various mechanisms, including paracrine signaling and direct neuron‐to‐glioma glutamatergic synapses, driving tumor progression [3, 4, 5, 8, 9].

Recent studies have expanded this view, revealing a more complex picture where GABAergic neurons can also exhibit tumor‐promoting effects, either through depolarizing GABAergic synapses in DMG [10], or by alternative mechanisms likely involving metabolic or paracrine interactions rather than synaptic transmission in GB [13]. It is conceivable that GB cells may also utilize GABA as a nutrient, as reported in medulloblastoma [53]. Moreover, given that inhibition of GABA catabolism with vigabatrin reduces brain metastasis [54], this strategy warrants investigation in the context of GB treatment. Notably, while glutamatergic signaling remains a dominant driver of neuron–glioma communication in GB, GABAergic pathways represent an important and previously unrecognized axis of glioma progression that needs further exploration.

Epigenetic and chromatin rewiring underpin the remarkable capacity of glioma cells to integrate into neural circuits [11, 12, 13]. However, the mechanisms by which neural cues are decoded at the genomic level to promote a pro‐tumorigenic response have only begun to be elucidated [11, 13]. Viewing neuron–glioma interactions through the epigenetic lens has opened new avenues for understanding how glioma cells exploit neural signals to their advantage. Importantly, modulating TFs mediating neuron–glioma communication can influence tumor growth [11]. Together, these insights call for exploration of new strategies targeting the epigenetic machinery that facilitates neuron–glioma interactions, with the aim of disrupting the neural circuitry that sustains glioma progression.

## Author Contributions

C.C. and I.N. drafted the initial manuscript and figures. S.R. conceived the idea and hypothesis, and prepared the final manuscript.

## Conflicts of Interest

The authors declare no conflicts of interest.

## Data Availability

Data sharing not applicable to this article as no datasets were generated or analyzed during the current study.
